# Phenolic Characterization of a Purple Maize (*Zea mays* cv. “Moragro”) by HPLC–QTOF-MS and Study of Its Bioaccessibility Using a Simulated In Vitro Digestion/Caco-2 Culture Model

**DOI:** 10.1021/acs.jafc.3c08960

**Published:** 2024-03-14

**Authors:** Marianela Desireé Rodriguez, Luisina Monsierra, Pablo Sebastián Mansilla, Gabriela Teresa Pérez, Sonia de Pascual-Teresa

**Affiliations:** † Department of Metabolism and Nutrition, Institute of Food Science, Technology and Nutrition (ICTAN), Consejo Superior de Investigaciones Científicas (CSIC), Jose Antonio Novais 10, Madrid 28040, Spain; ‡ Facultad de Ciencias Agropecuarias, Universidad Nacional de Córdoba, Córdoba 5000, Argentina; § Instituto de Ciencia y Tecnología de los Alimentos Córdoba (ICyTAC), Consejo Nacional de Investigaciones Científicas y Técnicas (CONICET)—UNC. Córdoba 5016, Argentina

**Keywords:** anthocyanins, *Zea mays* L, purple maize flour, antioxidant activity

## Abstract

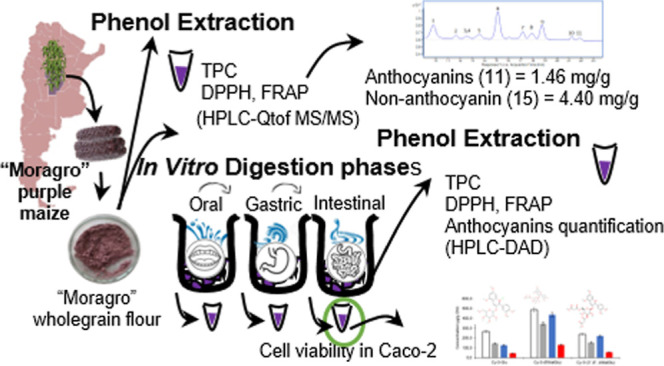

The present work aimed to characterize the phenolic and antioxidant content of the Argentinian purple maize “Moragro” cultivar. Additionally, the INFOGEST simulated in vitro digestion model was used to establish the effect of digestion on bioactive compounds. Finally, digestion samples were used to treat Caco-2 cells in the transwell model to better understand their bioavailability. Twenty-six phenolic compounds were found in purple maize cv. “Moragro”, 15 nonanthocyanins and 11 anthocyanins. Several compounds were identified in maize for the first time, such as pyrogallol, citric acid, gallic acid, kaempferol 3-(6″-ferulylglucoside), and kaempferol 3-glucuronide. Anthocyanins accounted for 24.9% of total polyphenols, with the predominant anthocyanin being cyanidin-3-(6″ malonylglucoside). Catechin-(4,8)-cyanidin-3,5-diglucoside and catechin-(4,8)-cyanidin-3-malonylglucoside-5-glucoside were detected as characteristics of this American maize variety. Total polyphenol content (TPC; by the Folin–Ciocalteu method), HPLC-DAD/MSMS, and antioxidant activity [by DPPH and ferric-reducing antioxidant power (FRAP)] were evaluated throughout in vitro digestion. TPC, DPPH, and FRAP results were 2.71 mg gallic acid equivalents (GAE)/g, 24 μmol Trolox equiv/g, and 22 μmol Trolox eq/g, respectively. The in vitro digestion process did not cause significant differences in TPC. However, the antioxidant activity was significantly decreased. Moreover, the bioavailability of anthocyanins was studied, showing that a small fraction of polyphenols in their intact form was conserved at the end of digestion. Finally, a protective effect of digested maize polyphenols was observed in the Caco-2 cell viability. The results suggest that “Moragro” purple maize is a good source of bioavailable anthocyanins in the diet and an interesting source of this group of compounds for the food industry.

## 1 Introduction

Maize (*Zea mays* L.) is a valuable crop with nutritional, cultural, environmental, and economic impacts in most countries in the world.^1^ Regarding its nutritional importance, maize is an excellent source of carbohydrates; it is naturally gluten-free, suitable for people suffering from celiac disease, and has special phytochemical components that can be beneficial for human health.^2^ This crop has great agronomical diversity, with different shapes and colors of grains ranging from white to yellow, red, blue, and purple. Besides, there are different maize varieties characterized according to the final use for which they are intended and their quality or the structure and composition of the grains. In this sense, the development of new germplasm is a great opportunity for the diversification and differentiation of this crop in the market with potential use for the food industry.^3^

Purple maize has been widely cultivated and consumed in the Andean and South American regions, mainly in Peru, Bolivia, Ecuador, and some regions of northern Argentina.^2^ In Argentina, the predominantly semiarid climate in the central area of the country limits maize production; therefore, one of the main genetic improvement efforts has been based on the development of adapted cultivars to these specific conditions.^4^ The Special Maizes Program at the Universidad Nacional de Córdoba focuses on the introduction, adaptation, and characterization of pigmented maize germplasm in the central semiarid region of Argentina. A new cultivar of purple maize (“Moragro”) has been obtained within this breeding program, which was registered for the first time in the country at the *Instituto Nacional de Semillas* (INASE). The commercial maize types grown in Argentina are traditional hybrid varieties; however, “Moragro” is an open-pollinated variety, which is characterized by minimizing dependence on external seed sources without causing yield losses and reducing the cost of production in agricultural systems.^5^ Moreover, it is a nontransgenic variety, adapted to late sowing dates for the central region of Argentina (late December/early January). Tolerance to semiarid climates is a distinctive feature of this cultivar, which performs well under rainfed conditions.^4^ Moreover, recent studies have shown that “Moragro” maize flour can be used as a functional ingredient in gluten-free bread, increasing its total polyphenol content (TPC), total anthocyanins, and antioxidant capacity. The flour also has a higher content of slowly digestible and resistant starch in comparison to traditional white maize flour. These findings suggest that “Moragro” maize has the potential to be a valuable crop for both human health and sustainable agriculture.^6^

Purple maize is a rich source of phenolic compounds, mainly anthocyanins, that give a dark purple-red color to the grains. The anthocyanin composition of some purple maize has been well studied. The 6 major anthocyanins include cyanidin-3-glucoside, pelargonidin-3-glucoside, peonidin-3-glucoside, and their malonic acid derivatives.^7^ Among minority compounds, condensed flavanol-anthocyanin pigments have been detected in purple maize from Peru and Mexico and can influence color, produce a darker red color, and might have stability advantages.^8^

Anthocyanins have a role in human health. Some benefits have been shown in diabetes, obesity, and cardiovascular disease.^9^ The health benefits of purple maize anthocyanins and other phenolic compounds depend on their bioavailability. During digestion, phenolics can undergo enzymatic and chemical modifications due to the different pH values of the medium.^10^ Moreover, some anthocyanins such as cyanidin-3-glucoside and pelargonidin-3-glucoside could be absorbed in their intact form in the gastrointestinal tract.^10^ Other anthocyanins and phenolics can reach the large intestine in significant amounts and undergo metabolism by the gut microbiota.^11^ In this sense, investigating the bioavailability of non-nutrients is a challenge for food technology due to the different mechanisms of their absorption and the often-complex nature of bioactive compounds.^12^ However, few studies have focused on the health effects of purple maize phenolic compounds.

Nowadays, our interest is in studying the nutritional and technological quality of grains and flour of the “Moragro” cultivar to produce healthy foods based on their nutraceutical properties. The aim of the present work was to investigate the phenolic composition of whole-grain purple maize “Moragro” from Argentina using high-performance liquid chromatography–quadrupole time-of-flight tandem mass spectrometry (HPLC–QTOF-MSMS), as well as the TPC and its antioxidant capacity. Additionally, their accessibility for absorption and their content after in vitro digestion were studied. Finally, the bioactivity of the phenolic compounds was determined through a Caco-2 model. The results of this study may contribute to a better understanding of the composition of the bioactive compounds of “Moragro” and their bioavailability.

## 2 Materials and Methods

### 2.1 Genetic Material and the Adaptation Process

The “Moragro” cultivar was obtained by crossing introduced genetic material from different origins: northern Argentina, Peru, Bolivia, and International Maize and Wheat Improvement Center (CIMMYT) seeds. The original population obtained was planted and assessed for five cycles (2011/12, 2013/14, 2014/15, 2015/16, and 2016/17) and the variety was stabilized in 2019. The adaptation process was carried out in each cycle through adaptive mass selection at the experimental station of the *Facultad de Ciencias Agropecuarias, Universidad Nacional de Córdoba*, Argentina (geographical location: 31°28′ 49.42″ S, 64°00′ 36.04″ W). The field is located in the central semiarid region of the country in the province of Córdoba, with an altitude of 425 m.a.s.l. The soil is Entic Haplustoll and presents a silt-loam texture on the superficial horizon. It is slightly acidic to neutral and well supplied with organic matter (Ministerio de Agricultura y Ganadería 2019). The field zone has a historical average range of medium, minimum, and maximum temperatures of 15–20, 8–13.7, and 21.8–25.1 °C, respectively, and an annual precipitation of 300 to 1000 mm (Bolsa de Cereales de Córdoba 2016). The grains used in the present work were obtained from the 2020/21 cycle.

### 2.2 Flour Obtention

The grains of purple maize (*Z. mays* L.), the “Moragro” cultivar, were milled on a cyclonic mill (Cyclotec CT193, Foss, Suzhou) to a fine powder (particle size range less than 500 μm). The whole-grain maize flour obtained was stored in darkness at −20 °C until chemical analysis.

### 2.3 “Moragro” Flour Characterization (Proximate Composition)

The moisture, protein, lipid, and ash contents of the “Moragro” whole-grain flour were measured according to the AACC methods (AACC International 2010)^13^ and expressed as g/100 g of flour of dry weight (DW). All analyses were performed in duplicate.

### 2.4 Characterization of Phenolic Compounds of “Moragro” Flour

#### 2.4.1 Phenol Extraction

Sample extraction to identify the initial compounds present in the “Moragro” flour was performed using the method of Chamorro et al.^14^ with modifications. In brief, 50 mg of sample was suspended in 1 mL of methanol/water (50:50 v/v acidified with formic acid 0.1%). The mixture was vortexed and sonicated for 15 min and then centrifuged at 10000 rpm for 15 min at 4 °C. The supernatant was collected, and the residue was re-extracted twice with 0.5 mL of acidified MeOH/H_2_O (1:1, 0.1% formic acid) following the same method and re-extracted following the same procedure two times. Supernatants were combined, filtered (0.45 μm), and stored at −20 °C until analysis.

#### 2.4.2 Total Polyphenol Content

TPC of the purple maize flour was carried out by the Folin–Ciocalteu reagent method, in the extracts obtained in the previous section, according to Silván et al.^15^ Gallic acid was used as the standard for the calibration curve. The absorbance was recorded at 725 nm in a BioTek Synergy HT multimode microplate reader (BioTek Instruments Inc., Winooski, VT, USA). Results were expressed as milligrams of gallic acid equivalents per gram of sample on a dry basis (mg of GAE/g of DW).

#### 2.4.3 Antioxidant Activity

Antioxidant activity was assessed as antiradical activity and ferrous-reducing power. Radical-scavenging capacity was measured using the DPPH method, as reported by Puell and de Pascual-Teresa.^16^ The ferric-reducing antioxidant power (FRAP) assay was performed using the protocol of Soriano-Maldonado et al.^17^ All samples were measured in triplicate using Trolox (Sigma-Aldrich) as the standard. Results were expressed as μmol of Trolox equivalents per grams of sample (μmol of Trolox eq/g DW).

#### 2.4.4 Identification and Quantification of Phenolic Compounds Using HPLC–QTOF-MS

The identification and quantification of “Moragro” purple maize phenolic compounds, including anthocyanin compounds, were performed using HPLC with mass spectrometry detection (Agilent 1200, Agilent Technologies) comprising a quaternary pump (G1311A), a diode array detector (Agilent G1315B), and a C18 analytical column (Phenomenex Luna, 3 μm, 4.6 mm × 150 mm) set thermostatically at 25 °C. The mobile phase consisted of water/formic acid, 99.9:0.1 v/v (solvent A), and acetonitrile/formic acid, 99.9:0.1 v/v (solvent B). The flow rate was kept at 0.5 mL/min. The gradient program was as follows: 90% A/10% B, 0–30 min; 70% A/30% B, 30–35 min; 65% A/35% B, 35–45 min; 60% A/40% B, 45–50 min; and 90% A/10% B, 50–60 min^18^ The injection volume was 5 μL for all samples and standards. Peaks were identified by comparing their retention time with the corresponding standards. For mass spectrometric analysis, an Agilent 6530 Accurate-Mass QTOF LC/MS with electrospray ionization (ESI) and Jet Stream technology (Agilent Technologies) operated at 325 °C was used. The capillary voltage and nebulizer gas flow were set to 4000 kV and 45 psi, respectively. Nitrogen was used as the drying gas at a flow rate of 8 L/min. The fragmented ions of the analytes were detected in positive and negative modes to provide extra certainty in the determination of the molecular masses. For the identification and quantification of compounds, MS and MSMS fragmentation spectra experiments were performed, and spectral signal data were also acquired at 280, 320, and 520 nm. For MSMS experiments, a quite generic collision energy of 20 V was used, as a compromise, to simplify the development of the method and ensure good fragmentation of the majority of targeted compounds. Data acquisition and processing were performed with Masshunter Data Acquisition (B.05.01) and Masshunter Qualitative Analysis (B.07.00 SP2) software. Compounds were identified by comparing mass spectra and retention time with the corresponding standard, if available. In the case of compounds for which standards were not available, identification was based on a prediction of chemical formula from accurate ion mass measurement and confirmed by comparing MSMS with data provided by relevant literature references (see Table 1). The analytical method was validated for all quantified compounds, with a minimum recovery of 85%, a minimum detection limit of 0.01 μg/mL, and a minimum quantification limit of 0.05 μg/mL for each quantified compound. The quantification was performed by interpolation into the calibration curve of an identical standard or a structurally related compound used to quantify it (equivalent) and expressed as μg per g of DW sample as follows: cyanidin-3-*O*-glucoside was used for the quantification of cyanidin derivatives, pelargonidin derivatives, and anthocyanin condensed forms; peonidin-3-glucoside was used as standard for peonidin derivatives; caffeic acid for cinnamoyl-quinic acids, gallic, ferulic, and citric acids; 3 caffeoylquinic acid for chlorogenic acid; quercetin-3-*O*-glucoside for quercetin-3-*O*-glucoside; quercetin for quercetin derivatives and morin; kaempferol for kaempferol derivatives; epicatechin for epicatechin and naringenin; apigenin for vitexin; and phloroglucinol for pyrogallol.

**Table 1:** Characterization of the Individual Phenolic Compounds in Moragro Flour Extracts Using HPLC–QTOF-MS

peak	compound assignment^b^^,^^c^	Rt^a^(min)	[M]^−^ identified	MS/MS^–^	[M]^+^ identified	MS/MS^+^
1	pyrogallol	2.9			127.0396	81, 53
2	citric acid	4.1	191.0198	111		
3	catechin-(4,8)-cy-3,5-diGlu	4.9			899.2250	737, 575, 423, 329, 287
4	gallic acid	5.3	169.0453	125		
5	catechin-(4,8)-Cy-3-MalGlu-5Glu	8.2			985.2241	823, 737, 575, 423, 329
6	Cy-3-Glu	9.9			449.1099	287
7	Pg-3-Glu	11.7	431.1025	269	433.1112	287
8	Cy-3-MalGlu	12.5			535.1075	449, 287
9	chlorogenic acid	12.5	353.0873	191		287
10	Pn-3̅-Glu	12.7			463.1240	301
8	Cy-3-MalGlu	13.6			535.1075	449, 287
8	Cy-3-(6′MalGlu)	15.2			535.1070	449, 287
11	caffeic acid	15.6	179.0358	135		
12	Pg-3,6-MalGlu	17.2			519.1123	433, 271
13	*Pn*-3̅,6-MalGlu	17.9			549.1229	463, 301
14	Cy-3-(diMalGlu)	18.0			621.1084	535, 449, 287
14	Cy-3-(3,6-diMalGlu)	18.8			621.1092	535, 449, 287
15	Pg-3,6-diMalGlu	21.2			605.1136	519, 433, 271
16	*p*-coumaric acid	21.4			165.0581	147, 45
17	Pn-3,6-diMalGlu	21.9			635.1136	549, 463, 301
18	quercetin-3-rutinoside	22.3	609.1507	301	611.1606	
19	ferulic acid	23.6			195.0636	176, 144
20	quercetin-3-Glu	23.8	463.1002	301	465.1019	
21	kaempferol 3-(6″-feruloylglu)	24			625.1549	287
22	kaempferol-3-Glu	27.2			449.1082	287
23	vitexin	35.2			433.1723	283
24	kaempferol-3-glucuronide	36.0			463.1826	287
25	naringenin	43.1			273.0763	189, 153
26	morin	43.6	301.0718	149		

a RT, retention time.

b Identification was confirmed according to the standard (Std) above cited and/or the MS fragmentation pattern previously described by other studies.

c Cy, cyanidin; Glu, glucoside; Mal, malonyl; Pg, pelargonidin; Pn, peonidin.

### 2.5 Bioaccessibility of Phenolic Compounds in “Moragro” Flour

#### 2.5.1 Static In Vitro Digestion

The purple maize “Moragro” flour was in vitro digested using the INFOGEST protocol^19^ with oral (pH 7), gastric (pH 3), and intestinal (pH 7) phases. The enzymes used for each gastrointestinal phase were salivary amylase (75 U/mL), pepsin (2000 U/mL), pancreatin (100 U trypsin/mL), and porcine bile extract (10 mM). A control tube lacking the flour served as a digestion blank. Following this, oral and gastric aliquots (0.5 mL) were collected. The intestinal digest was centrifuged (5000 rpm, 10 min), and its supernatant was collected in 2 mL Eppendorf tubes. All samples were stored at −20 °C until analysis.

#### 2.5.2 Characterization of Digested Aliquots

##### 2.5.2.1 Phenol Extraction, TPC, and Antioxidant Activity

Polyphenol extraction from digested samples was performed as mentioned above in Section 2.4.1 with one modification. For the first step, 0.5 mL aliquots were taken at each digestion phase, placed in an Eppendorf, and added with 0.5 mL of methanol (acidified with formic acid, 0.1%). Then, the procedure continued as described before. TPC was determined as described in Section 2.4.2, and antioxidant activity was determined according to Section 2.4.3.

##### 2.5.2.2 Quantitative Analysis of Anthocyanins

Quantitative analysis of anthocyanins before (in the undigested “Moragro” flour), during, and after digestion was carried out using an Agilent 1200 series liquid chromatograph with a quaternary pump and a photodiode array detector equipped with a Phenomenex Luna C18 column (3 μm; 4.6 × 150 mm) set at 25 °C. Aqueous 0.1% formic acid (solvent A) and 0.1% formic acid acetonitrile (solvent B) were used at a flow rate of 0.5 mL/min. We started with 90% A/10% B, 0–30 min to 68% A/32% B, 30–35 min to 62% A/38% B, and 35–40 min to 53% A/47% B, followed by an additional 5 min isocratically at 47% B and 10 min column stabilization at 10% B prior to the next analysis. Anthocyanins were detected at 520 nm, and their peak areas were referred to a calibration curve obtained with cyanidin-3-glucoside. Limits of detection and quantification were calculated and were in every case below 0.1 μg/mL.

#### 2.5.3 Bioactivity Analysis

##### 2.5.3.1 Cell Culture and Differentiation

Cell culture and differentiation were performed following the protocol reported by Hubatsch et al.^20^ In brief, before their use in this assay, Caco-2 cells were cultured in culture flasks containing Dulbecco’s modified minimal essential medium (DMEM) supplemented with 10% heat-inactivated fetal bovine serum (FBS), nonessential amino acids (1%), and 1% antibiotic (streptomycin/penicillin) solution at 37 °C and in a humidified atmosphere with 5% CO_2_. The medium was replaced every 2 days. Cells were subcultured weekly upon 85–95% confluence by trypsinization. Caco-2 cells were used in a maximum passage of 60 and seeded into 24-well *trans*-wells at a concentration of 6 × 10^5^ cells/mL in DMEM with 10% FBS, nonessential amino acids (1%), and 1% antibiotic (streptomycin/penicillin). The medium was changed in the apical (150 μL) and basolateral (700 μL) chambers every 2 days. The *trans*-epithelial electrical resistance values were measured to confirm monolayer formation and cell differentiation.

##### 2.5.3.2 Cell Viability

The (3-[4,5-dimethylthiazol-2-yl]-2,5 diphenyl tetrazolium bromide) (MTT) assay was used to determine the cell viability. Caco-2 cells were plated in 96-well plates (1.6 × 10^6^ cells/mL) and cultured for 7 days at 37 °C in 5% CO_2_ for differentiation. The differentiated Caco-2 cells were treated with diluted intestinal digestion aliquots in the following proportions: 1:1, 1:10, 1:100, 1:250, 1:500, and 1:1000, all of them suspended in serum-free DMEM. The medium was removed after 18 h. The cells were sequentially washed with phosphate buffered saline (PBS), which was then removed, 200 μL serum-free DMEM was added, and 20 μL of an MTT solution (5 mg/mL in PBS) was added to each well and incubated for an additional 2 h at 37 °C in 5% CO_2_. Formazan crystals formed in the wells were solubilized in 200 μL of DMSO (dimethyl sulfoxide). The measurement was performed with an absorbance at a 570 nm wavelength employing a microplate reader (PowerWaveTM XS) in a UV spectrophotometer (BioTek Instruments, Inc., Winooski, VT, USA). The assay was repeated in two independent experiments. The viability was calculated in comparison to control experiments in which a solvent control was added in place of polyphenols, and that was used as a 100% viable reference.^18^ Dilution was performed with one-part intestinal aliquot and one-part DMEM (sample: DMEM). The control sample consisted of an intestinal aliquot of the digestion blank (purple maize flour was replaced with water, and in vitro digestion was performed).

### 2.6 Statistical Analysis

The results were expressed as the mean of two replicates ± the standard deviation. Analysis of variance was performed, and data were compared by the DGC means-comparison test^21^ with a significant level at 0.05. These analyses were performed using Infostat Statistical Software (Facultad de Ciencias Agropecuarias, UNC, Argentina).

## 3 Results and Discussion

### 3.1 “Moragro” Flour Characterization

#### 3.1.1 Proximate Composition

The macronutrient composition of “Moragro” flour obtained was 1.85 ± 0.03% ash, 6.1 ± 0.1% lipids, and 10.2 ± 0.2% protein. In comparison with other maize varietal types, the “Moragro” cultivar presented higher protein, lipid, and ash contents than those of blue and white maize flour from México.^4,22^ “Moragro” flour presented higher protein and lipid contents and lower ash content than that of several purple maize genotypes from India.^23^ As maize flour is generally rich in antioxidant compounds and starch, it is ideal for the development of functional foods.^24^

#### 3.1.2 TPC and Antioxidant Activity

The extractable TPC of “Moragro” flour was 2.71 ± 0.04 mg GAE/g DW. The antioxidant potential of “Moragro” flour was 24 ± 1 μmol of Trolox eq/g DW assessed by analyzing its antiradical activity (DPPH) and 22 ± 1 μmol of Trolox eq/g DW by FRAP. Polyphenols exhibit antioxidant capacity and act as free radical inhibitors; we established significant correlations (*r*) between TPC and DPPH (*r* = 0.76, *p* < 0.05) and DPPH and FRAP (*r* = 0.80, *p* < 0.01), suggesting a direct relationship between polyphenol content and antioxidant activity.

“Moragro” maize presented higher TPC in comparison to that of white and yellow maize from India, with a value of 1.6 mg GAE/g and 1.3 mg GAE/g of TPC, respectively.^23^ This higher polyphenolic content of purple maize could be expected because it contains anthocyanins in addition to ferulic and *p*-coumaric acids that have been detected in white maize.^25^

In comparison with the blue maize flour from Mexico, “Moragro” flour showed lower TPC but higher antioxidant activity (DPPH, FRAP) than it.^22^ In addition, “Moragro” flour presented lower antiradical activity than that reported by Ranilla et al.^26^ for a Peruvian variety of purple maize ″Canteño″. On the other hand, in a variety of purple waxy maize (var. “Ceratina”) from Thailand, the value of TPC and ferric-reducing power were similar to our results obtained.^27^

These results indicated that “Moragro” flour is an important source of phenolic compounds with antioxidant activity.

#### 3.1.3 Characterization of the Composition by HPLC–QTOF-MS

The phenolic compounds identified by HPLC–QTOF-MS analysis in “Moragro” flour are shown in Table 1. A total of 26 compounds were identified: 15 nonanthocyanin and 11 anthocyanin pigments. For practical reasons, we have classified the compounds into anthocyanins and nonanthocyanins for further analysis and description. Also, the phenolic compounds identified were quantified, and the results are shown in Tables 2 and 3.

**Table 2:** Nonanthocyanin Content of Moragro Flour

compound assignment	concentration^a^ (μg/g)
**benzoic acids**	
citric acid	755.7 ± 60.4
gallic acid	1.3 ± 0.1
chlorogenic acid	6.6 ± 0.5
**cinnamoyl-quinic acids**	
caffeic acid	1296.8 ± 103.7
*p*-coumaric acid	6.5 ± 0.2
ferulic acid	6.0 ± 0.4
**flavonols**	
quercetin-3-rutinoside	588.0 ± 29.4
quercetin-3-Glu	14.7 ± 0.6
kaempferol 3-Glu	25.8 ± 1.5
kaempferol 3-glucuronide	1201.0 ± 9.2
morin	11.5 ± 0.7
kaempferol 3-(6″-feruloylGlu)	nq
**other compounds**	
naringenin	2.0 ± 0.1
vitexin	254.5 ± 12.7
pyrogallol	229.4 ± 13.8
total nonanthocyanin compounds	4399.9 ± 395.9

a Average value ± the standard deviation (*n* = 3).

**Table 3:** Anthocyanin Content of Moragro Flour^a^

peak	compound assignment	concentration (μg/g)
	catechin-(4,8)-Cy-3,5diGlu	15.9
	catechin-(4,8)-Cy-3-MalGlu-5Glu	9.0
1	Cy-3-Glu	314.3
2	Pg-3-Glu	115.7
3	Cy-3-(MalGlu)	4.2
4	*Pn*3̅-Glu	142.6
5	Cy-3-(MalGlu)	68.1
6	Cy-3-(6′MalGlu)	356.5
7	Pg-3-(6′MalGlu)	108.3
8	*Pn*3̅-(6′MalGlu)	123.4
9	Cy-3-(diMalGlu)	8.9
9	Cy-3-(3″,6″, diMalGlu)	107.3
10	Pg 3-*O*-3′,6′-*O*-diMalGlu	41.3
11	Pn 3-*O*-3′,6′-*O*-diMalGlu	44.8
	total anthocyanin concentration	1460.4
	cyanidin derivatives	859.3
	peonidin derivatives	310.8
	pelargonidin derivatives	265.3
	condensed forms	24.9

a Cy, cyanidin; Glu, glucoside; Mal, malonyl; Pg, pelargonidin; Pn, peonidin.

##### 3.1.3.1 Nonanthocyanin Compounds

The 15 nonanthocyanin compounds identified in the “Moragro” cultivar (Table 1) were classified according to their structure as benzoic acids, cinnamoyl-quinic acids, flavonols, and other phenolic compounds (Table 2).

Benzoic acid derivatives were recognized as citric, gallic, and chlorogenic acids. Peak 2 to be assigned citric acid was identified from their precursor ion [M – H]^−^ at *m*/*z* 191. Gallic acid was presented by peak 4 with a molecular ion of [M – H]^−^ at *m*/*z* = 169. Chlorogenic acid (peak 9) was identified by comparison of its molecular mass ion of [M – H]^−^ at *m*/*z* 353 and retention times of 12 and 5 min with the data obtained with the commercial standard.

Peaks 11, 16, and 19 were identified as cinnamoyl-quinic acids based on data from a previous survey^14^ and their precursor ion fragments at [M – H]^−^ 179 and [M + H]^+^ 165 and 195, corresponding to caffeic, *p*-coumaric, and ferulic acids, respectively. Caffeic acid identification was further confirmed using a commercial standard.^14^

Other compounds of peaks 18, 20, 21, 22, 23, and 26 were identified as flavonol derivatives from kaempferol and quercetin. Compound 20 was negatively identified as quercetin-3-glucoside at *m*/*z* 452 and positively at *m*/*z* 465 by comparison with the corresponding commercial standard. Compounds 18 and 26 were negatively identified at *m*/*z* 609 and 301 as quercetin-3-rutinoside and morin according to a previous investigation.^18,28^ Peak 22 was identified as kaempferol-3-glucoside with a molecular ion at *m*/*z* 449. Peaks 21 and 24 were named kaempferol 3-(6″-feruloylglucoside) and kaempferol-3-glucuronide with a molecular ion at *m*/*z* 625 and 463 with a typical fragment of 287 corresponding to kaempferol, the free aglycone moiety, resulting from the loss of the glucose and feruloylglucose, respectively. Kaempferol 3-*O*-glucuronide has been identified in different fruits such as *Sarcandra glabra*,^29^ berries of the Rosaceae family,^30^ and strawberry,^31^ but it has not been identified or quantified in purple maize until now. The same happens with kaempferol 3-(6″-feruloylglucoside), which has only been reported so far in *Polylepis incana*.^32^

Furthermore, other compounds were identified as pyrogallol, vitexin (flavone), and naringenin (flavanone) with molecular ions with *m*/*z* values of 127, 433, and 273, respectively. Pyrogallol was identified as reported by Hidalgo et al.^33^ Naringenin and vitexin were confirmed with data from Chatham et al.^34^

The nonanthocyanin compounds found in this work are characteristic of purple maize, and HPLC–MSMS fragmentation of their main compounds has been described by Paucar-Menacho et al.^35^ Likewise, these compounds were identified, characterized, and quantified in other matrixes in previous studies by the group.^14,18,33^ Additionally, other reports by Gálvez Ranilla et al.,^36^ Del Pozo-Insfran et al.,^37^ and Ramos-Escudero et al.^28^ provide data on the quantification of some phenolic compounds in purple maize. All of these reports have been consulted to confirm the identity of the nonanthocyanin compounds in this research. However, the presence of pyrogallol, citric acid, gallic acid, kaempferol 3-(6″-feruloylglucoside), and kaempferol 3-glucuronide has not been previously reported in purple maize, resulting in compounds distinctive to the “Moragro” cultivar.^28,35−38^

Regarding quantification, the results showed differences between the amount of nonanthocyanin and anthocyanin compounds (Tables 2 and 3). “Moragro” flour found a total of 4399.9 μg/g DW of nonanthocyanin compounds, presenting 75.1% of total phenol compounds quantified, and the majority main compound was free caffeic acid with 22.1%, and the second major compound was kaempferol 3-*O*-glucuronide that accounted for 20.5%. In contrast with our results, a previous study by Paucar-Menacho et al.^35^ presented a lower amount (323.9 μg/g DW) of nonanthocyanin compounds, and ferulic acid derivatives were the most dominant nonanthocyanin compound found in purple maize (*Z. mays* L. *var. PMV-581*). Furthermore, Ramos-Escudero et al.^28^ reported a lower concentration of caffeic acid on purple maize (INIA-601), and Gálvez Ranilla et al.^36^ reported a lower concentration of free caffeic acid and ferulic acid, which was notably higher than in our study. On the other hand, a more recent study showed that ferulic acid is the most abundant phenolic acid in purple maize from Mexico.^25^

Cinnamoyl-quinic acids represented a percentage of 22.3%, flavonols accounted for 31.4%, and benzoic acids accounted for 13.1% of total polyphenol compounds. The remaining percentage of total polyphenol compounds (8.3%) consisted of naringenin, vitexin, and pyrogallol. The results obtained showed the presence of quercetin and kaempferol derivatives within the flavonol class in relevant quantities. Some of these compounds were identified by Paucar-Menacho et al.,^35^ and an interesting difference was the lower concentration of quercetin-3-rutinoside (35.91 μg/g) than that in “Moragro” flour (588.0 μg/g). Quercetin and kaempferol have been identified and quantified in several studies, but their derivatives have not been detailed yet, and in comparison, “Moragro” flour showed a higher concentration of quercetin derivatives than that reported.^2,28,39^ In addition, among benzoic acids, citric acid was present in “Moragro” flour with a 12.9% phenol total quantified, while in other purple maize, it has not been detected.

The “Moragro” cultivar was distinguished from the other purple maize varieties by the presence of pyrogallol, citric acid, gallic acid, kaempferol 3-(6″-feruloylglucoside), and kaempferol 3-glucuronide, as mentioned above. Furthermore, in the quantification, the “Moragro” cultivar was distinctive by showing relevant amounts of caffeic acid, kaempferol 3-*O*-glucuronide, citric acid, and quercetin-3-rutinoside as the four major constituents among the total phenols identified and quantified. On the other hand, previous studies showed that ferulic acid and its derivatives were the most dominant nonanthocyanin compounds in different purple maize varieties, while in our study, ferulic acid only represented 0.1% of the total phenols quantified, being another distinctive feature of this cultivar.

##### 3.1.3.2 Anthocyanins

Up to 11 anthocyanin pigments were identified in “Moragro” flour, particularly cyanidin (Cy), pelargonidin (Pg), and peonidin (Pn) derivatives (Table 1). The identity of each compound was elucidated by comparison to the commercial standard used. In this regard, peaks 6 at 9.9 min and 10 at 12.7 min were identified as cyanidin-3-glucoside and peonidin-3-glucoside, respectively, and peak 7 was identified as pelargonidin-3-glucoside by comparison of their retention time and spectrum mass with data in our library and previous studies. Acyl derivatives of cyanidin, peonidin, and pelargonidin were observed in peaks 8, 12–15, and 17, respectively. In addition, peak 8 matches a molecular ion at *m*/*z* 535, releasing the MS/MS^+^ fragment at *m*/*z* 449 ([M-86]^+^, loss of a malonyl residue) and at *m*/*z* 287 ([M-248]^+^, loss of malonyl glycoside moiety), corresponded with cyanidin-3-malonylglucoside. For peak 12, the molecular ion was at *m*/*z* 519 that released two fragments MS/MS^+^ at *m*/*z* 433 ([M-86] ^+^, loss of a malonyl residue) and at *m*/*z* 271 (pelargonidin), corresponded with pelargonidin-3,6-malonylglucoside. Also, peak 13 with a molecular ion 549, which releases two fragments MS/MS^+^ at *m*/*z* 463 ([M-86] ^+^, loss of a malonyl residue) and *m*/*z* at 301 (peonidin), corresponded with peonidin-3,6-malonylglucoside. Peaks 14, 15, and 17 were [M+86] ^+^ greater than that of peaks 8, 1, 2, and 13, respectively, and showed a similar fragmentation pattern, so they can be assigned, respectively, to cyanidin-3-(6′malonylglucoside), pelargonidin-3,6-malonylglucoside, and peonidin-3,6-malonylglucoside. Further confirmation of the identification performed was provided by a comparison of the same compounds with others previously identified in purple maize.^7,40,41^

In addition, two compounds involving the condensation of an anthocyanin unit (Cy) and catechin residues were also found. These compounds were identified as catechin-(4,8)-cyanidin-3,5 diglucoside (peak 3) and its acylated condensed form, catechin-(4,8)-cyanidin-3-malonylglucoside-5 glucoside (peak 5) by comparison with pigments with similar spectrum mass, fractions, and structural characteristics of Apache Red Purple Corn (Siskiyou Seeds, Williams, OR).^34^

**Figure 1:**
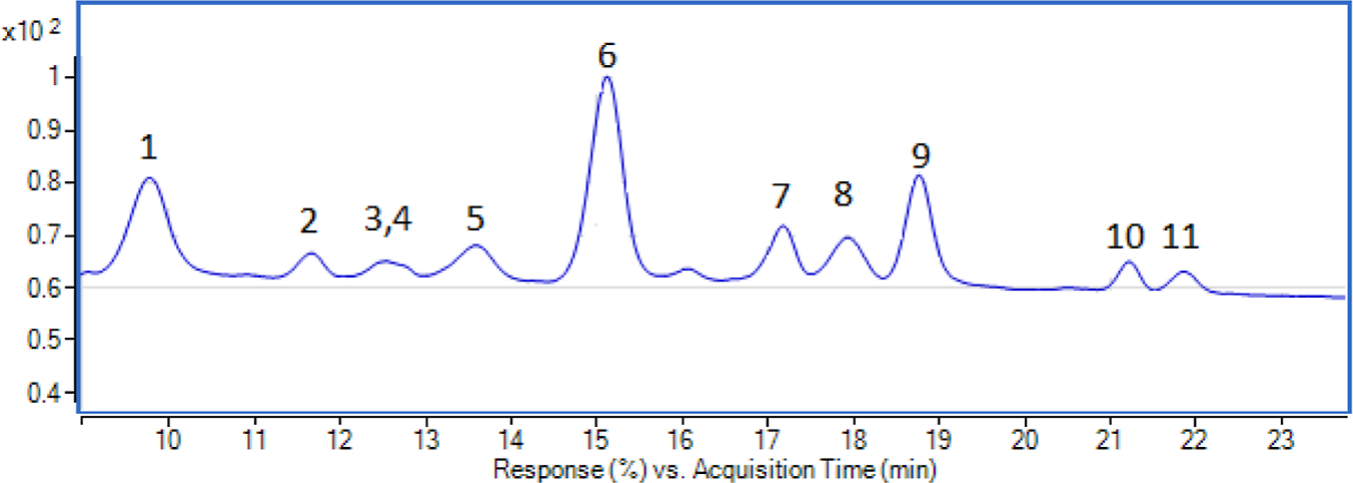
Anthocyanin compounds identified in Moragro maize flour extract at 520 nm.

An exhaustive identification and quantification were performed to the anthocyanin profile, the HPLC-DAD chromatogram of “Moragro” flour extract is shown in Figure 1, and the compounds were classified according to their anthocyanidin and quantified in Table 2. Anthocyanin compounds represent 24.9% (1460 μg/g) of total phenolic compounds with a high molecular diversity. The main anthocyanin compounds present in the “Moragro” cultivar were cyanidin derivatives, representing more than 58% of the total anthocyanin content. The prevalent anthocyanins were cyanidin-3-(6″ malonylglucoside), which accounted for 29%, and its respective nonmalonyl constituent, cyanidin-3-glucoside with 21% of the total anthocyanin content. The anthocyanin profile was in agreement with other studies with differences in the concentration and dominant compounds.

The total concentration of anthocyanin content was higher in purple maize from Peru (*Z. mays* L. *var. PMV-581*), accounting for 92% of the total phenolic compound with 3636.41 μg/g, than that in the “Moragro” cultivar, and in purple maize from Peru, Cy-3-Glu was the major anthocyanin.^35^ Also, Pedreschi and Cisneros-Zevallos^39^ reported that Cy-3-Glu was the major anthocyanin, constituting ∼38%, followed by the acylated cyanidin-3-glucoside with ∼26% of the total anthocyanin content. Although, in Apache red purple maize from the USA, the most abundant were pelargonidin derivatives (1400 mg/g).^42^ In agreement with our dominant anthocyanin, Camelo-Méndez et al.^43^ reported cyanidin-3-(6″ malonylglucoside) as the major anthocyanin. Furthermore, in black sweet maize from China, despite being a quite different variety, the total anthocyanin content was similar, but the main compounds were pelargonidin derivatives, followed by cyanidin and then peonidin derivatives.^44^ The differences in the major anthocyanins among the mentioned studies suggest that each purple maize variety had its own dominant anthocyanin type. Moreover, the dominant anthocyanins were related to plant pigment; in a study, the authors showed that while the predominant anthocyanins in blue-aleurone and purple-pericarp maize were cyanidin-based glucosides, pelargonidin-based glucosides were the dominant in reddish-purple-pericarp and cherry-aleurone accessions.^45^

The second place was for peonidin derivatives, accounting for 21% of the total anthocyanins. Among them, the major compound was peonidin-3-*O*-glucoside. Similarly, in Andean purple maize, peonidin derivatives were the second most abundant compound of total anthocyanins,^39^ whereas in other studies, peonidin derivatives were the minority compound anthocyanins.^8,44,45^

Third among total anthocyanins, pelargonidin derivatives were observed to reach 18%. Pelargonidin-3-glucoside was the most concentrated with 115.7 μg/g DW. The same compounds were identified in González-Manzano et al.,^8^ but in this study, the predominant compound was pelargonidin-3-(6″malonylglucoside).

As was mentioned, in “Moragro” flour were detected catechin-(4,8)-cyanidin-3,5 diglucoside and catechin-(4,8)-cyanidin-3-malonylglucoside-5 glucoside. These condensed pigments were present as minority compounds of the total anthocyanin derivatives with a 1.7% value. Flavanol anthocyanin condensed forms were also identified in many studies, being between 0.3 and 3.2% of condensed pigments, according to González-Manzano et al.^8^ In “Moragro” flour, only condensed versions containing cyanidin were identified, but it has been reported in Apache Red purple maize that other condensed forms of pelargonidin, or peonidin, and (epi)afzelechin have been identified by Chatham et al.^34^

The identification of flavanol-anthocyanin condensed compounds in our purple maize (var. “Moragro”) and various American varieties, including two distinct Mexican purple maize varieties (cv. Arrocillo and cv. Peruano),^8^ Peruvian purple maize cultivars (var. PMV-581),^35^ and Apache Red purple maize from the USA,^42^ underscores a potentially distinctive genetic trait shared among American germplasms of purple maize. This finding may serve as a genetic marker that differentiates them from their European counterparts. In contrast, European pigmented varieties of purple maize, such as “Millo Corvo”^46^ from Spain and “Moradyn”^38^ from Italy, have not been reported to exhibit the presence or identification of these condensed compounds, as far as current knowledge extends. This disparity in the occurrence of flavanol-anthocyanin compounds could potentially serve as a distinguishing feature between American and European germplasms of purple maize.

### 3.2 Survival of Polyphenols during In Vitro Digestion and Bioaccessibility

#### 3.2.1 Effect of In Vitro Digestion on TPC and Antioxidant Activity

The increase in research aimed at the study of polyphenols and their healthy properties has been a turning point in the field of food science. Numerous investigations have been carried out to determine the total content, antioxidant activity, and composition of polyphenols in a wide variety of foods. However, it is important to study how polyphenols get through the digestive process as this is fundamental to evaluating their health benefits. The digestion process induces changes in the food composition, including polyphenol content and antioxidant activity, and deserves to be investigated, so a study of the raw material (flour) is a fundamental step for producing food products.

The impact of in vitro digestion of “Moragro” purple maize flour on its TPC and antioxidant activity is shown in Table 4.

**Table 4:** Influence of In Vitro Digestion on TPC and Antioxidant Activity

assay	undigested matrix	oral phase	gastric phase	intestinal phase
TPC (mg GAE/g DW)	2.71 ± 0.04^b^	1.54 ± 0.03^a^	2.30 ± 0.10^b^	2.60 ± 0.30^b^
antiscavenging activity (μmol of Trolox eq/g DW)	24.0 ± 1.0^c^	8.3 ± 0.2^a^	9.0 ± 1.0^a^	15.1 ± 0.8^b^
reducing power (μmol of Trolox eq/g DW)	22.0 ± 1.0^c^	11.7 ± 0.6^b^	1.4 ± 0.4^a^	14.0 ± 2.0^b^

Different letters within a line indicate statistically significant differences in the DGS test (*p* < 0.05). DW: dry weight; GAE: gallic acid equivalent.

The oral phase induces a significant modification of the polyphenolic content, as attested by recovery rates of 57% by the Folin–Ciocalteu method compared to those of the undigested matrix (control). In the gastric and intestinal phases, there are no significant differences from the control. The antioxidant activity showed a significant decrease after oral phase digestion considering both mechanisms with a remaining activity of 34% for DPPH and 53% for FRAP according to a decrease in TPC during this in vitro digestion step. In contrast with TPC results, gastric conditions produced a significant decrease in antioxidant properties, which was observed compared to those of the undigested matrix (Table 4). The lowest antioxidant activity was obtained in the gastric phase, with a recovery rate of 38.8% for antiradical activity and 6.1% for reducing power. Finally, in the intestinal phase, 61.8% and 64.6% of the antiradical activity and reducing power, respectively, were retained compared to those of the control.

In the oral phase, TPC and antioxidant activity were lower than those in the undigested matrix, although saliva helps with polyphenol solubilization, which substantially increases their availability.^47^ Other factors such as the variation of pH, phenolic composition, and presence of enzymes (in this case, α-amylase)^48^ affect antioxidant activity. During the gastric phase, the pH was the lowest in the in vitro digestion process, which could protect some polyphenols against degradation, such as phenolic acids, flavonols, and anthocyanins, while other polyphenols can be destroyed as the flavonoids oligomers that degrade to smaller units.^49^ TPC did not show significant differences during the gastric phase compared with the control, but the antioxidant activity decreased. This can be explained by the fact that protonation or deprotonation reactions can occur when pH varies, and this affects the oxidative state and properties of the polyphenol’s compounds.^50^ The high recovery rate of the TPC after in vitro digestion tends to indicate that the very large majority of the products are still present, maybe indicating a liberation of the structure of polyphenols and, consequently, an increase in potentially bioaccessible compounds through in vitro digestion phases.^51^ However, the antioxidant activity was lower at the end of digestion compared with that of the undigested matrix. This fact could be mainly attributed to the high pH in the intestinal phase and the reactions that could occur such as deglycosylation, glucuronidation, methylation, sulphonation, and hydroxylation.^49^ The results of our study showed that in vitro digestion of purple maize flour affects antioxidant activity but retains about 60%, while the TPC remained without significant changes in comparison to that at the end of digestion with the undigested matrix, so “Moragro” flour will be an important raw material for elaborated products.

Despite extensive characterization, identification, and quantification of phenolic compounds of purple maize, only a few studies have examined the effect on compounds of purple maize after in vitro digestion.^38,43^ Compared with Ferron et al.^38^ TPC, mainly anthocyanins and flavonols, were detected by RP-HPLC-UV, and at the end of the digestion, all marker compounds reduced notably, while in our results, the TPC between the undigested and digested purple maize matrixes did not differ significantly.

An interesting study was conducted by Sęczyk et al.^52^ where the effect of in vitro digestion on the bioaccessibility of TPC of cereal flours (wheat, durum wheat, whole wheat, yellow maize, and white rice flour) has been well studied. In contrast to our results, they reported that in vitro digestion and combination with the food matrix had a negative effect on the TPC compared to their initial amount. Also, our results contrast with those reported by Méndez Lagunas et al.,^53^ who found a significant increment of TPC in the intestinal phase of blue maize tortillas, and the difference is due to the fact that their product was previously processed and cooked, while ours was raw flour. Concerning antioxidant activity, in purple flour, FRAP and ORAC values increased after in vitro digestion.^54^ Furthermore, a similar behavior was reported in cooked whole-wheat pasta when the phenolic content was higher and the antioxidant capacity was lower than those in its control.^55^

The contradictory results found in the different reports confirm that bioaccessibility is influenced by digestion conditions, pH, temperature, enzymes used, as well as the characteristics and composition of the food matrix, texture, and the synergistic or antagonistic effect of the macromolecules with the bioactive compounds.^53^ Another parameter influent is the type of polyphenol constituents; some families of polyphenols are more resistant than others. For example, proanthocyanidins (catechin-(4,8)-Cy-3,5-diGlu and catechin-(4,8)-Cy-3-MalGlu-5-Glu) have shown significant resistance to digestion due to their chemical structure, which allows them to resist enzymatic hydrolysis in the gastrointestinal tract. Specifically, the presence of C–C and C–O–C flavonoid bonds in their structure allows them to reach the large intestine intact, where they can be fermented by the gut microbiota and generate health-beneficial metabolites. This resistance to digestion may have implications for the bioavailability and physiological effects of these compounds in the human body. These condensed compounds have demonstrated greater retention in the human gastrointestinal tract and, therefore, greater absorption in the human body compared to that of other phenolic compounds^56^ or anthocyanins that have more resistance to in vitro digestion than that of other phenolic compounds due to the presence of glycosidic bonds in their chemical structure. These bonds are more difficult to hydrolyze by digestive enzymes compared to other ester or carbonate bonds present in other phenolic compounds. In addition, the position of hydroxyl groups in the molecular structure of anthocyanins may also contribute to their resistance to enzymatic digestion.^57^

#### 3.2.2 Effect of In Vitro Digestion on Anthocyanin Content

As anthocyanins can be absorbed intact despite their different molecular sizes and types of sugar or acylated groups attached, the static in vitro digestion method can provide interesting information about their bioaccessibility.

Anthocyanin extracts from each in vitro digestion phase (oral, gastric, and intestinal) were analyzed by HPLC-DAD to evaluate changes in the anthocyanin content and profile.

**Figure 2:**
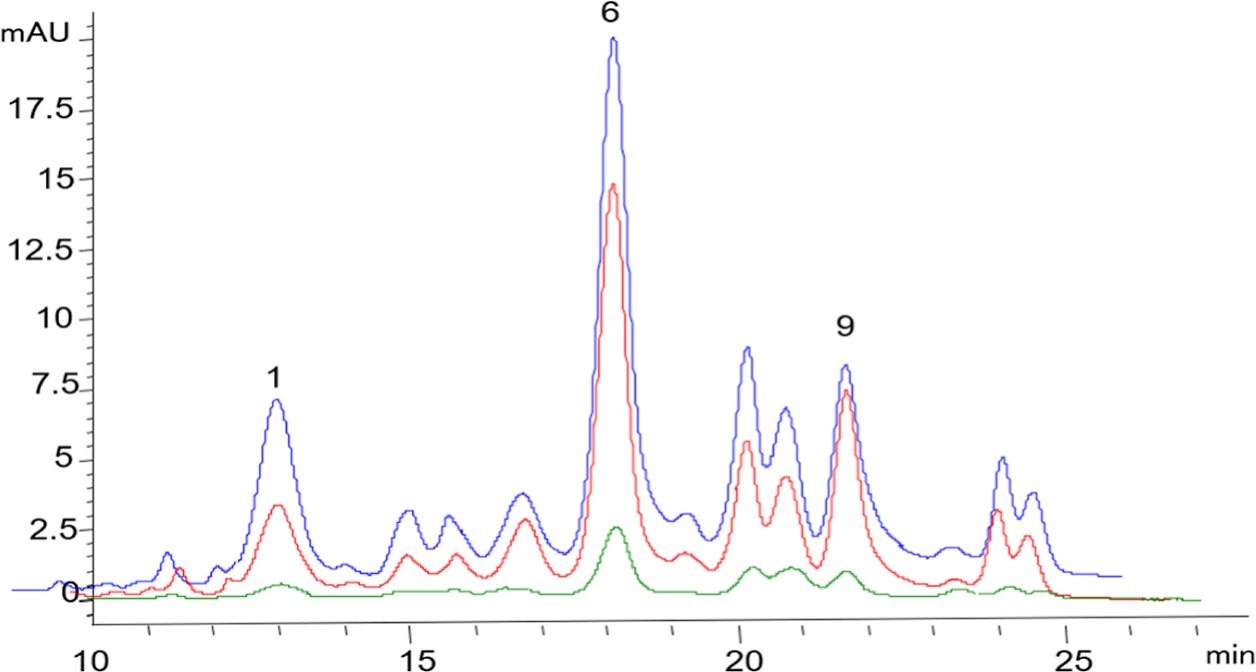
Anthocyanin profile of Moragro flour at 520 nm in the oral phase (blue), gastric phase (red), and intestinal phase (green). Peak 1, cyanidin-3-glucoside; Peak 6, cyanidin-3-(6′malonylglucoside); and Peak 9, cyanidin-3-(3″,6″, dimalonylglucoside).

The effect of digestion at each phase and on the different anthocyanins can be seen in Figure 2. Based on the HPLC–QTOF results (Table 1), three different marker compounds were selected in the sample and monitored during digestion. Cyanidins were the main compounds among anthocyanins, and cyanidin-3-glucoside (peak 1), cyanidin-3-(6′malonylglucoside) (peak 6), and cyanidin-3-(3″,6″, dimalonylglucoside) (peak 9) were chosen because they were present at the end of the digestion and can be differentiated from other signals. In general, at the end of the digestion, it can be seen which anthocyanins survived and which did not. There is a clear decrease in the peaks of each compound toward the end of the digestion, particularly in peaks that appear during the first 17 min that seem to disappear at the intestinal phase; Cy-3-Glu, Cy-3-(6″MalGlu), and Cy-3-(3″,6″, diMalGlu) were the major compounds at the end of the digestion, with the Cy-3-(6″MalGlu) being dominant.

**Figure 3:**
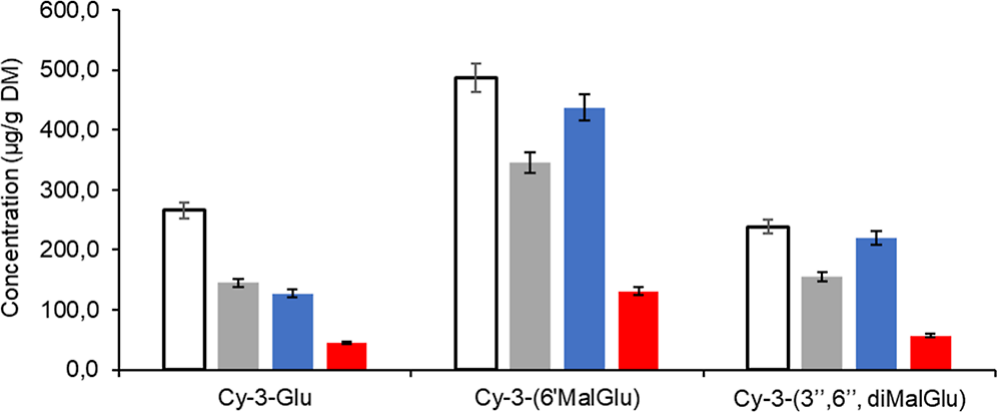
Marker anthocyanin content of Moragro flour at 520 nm in undigested matrix-control (white), oral phase (green), gastric phase (blue), and intestinal phase (red). Cy-3-Glu, Cy-3-(6″diMalGlu), and Cy-3-(3″,6″, diMalGlu) markers. Cy, cyanidin; Glu, glucoside; Mal, malonyl; Pg, pelargonidin; Pn, peonidin.

The quantification of three cyanidin derivatives is shown in Figure 3. The percentage recovery of the marker anthocyanins at each phase of in vitro digestion was calculated by comparing their concentration at a particular digestion phase with that of the undigested (control). In the oral phase, a decrease in the total content of the three cyanidin derivatives was observed, with the remaining 54% for cyanidin-3-glucoside (144.7 μg/g DW), 71% for cyanidin-3-(6″malonylglucoside) (345.5 μg/g DW), and 65% for cyanidin-3-(3″,6″, dimalonylglucoside) (155.5 μg/g DW). In contrast, during the gastric phase, an increase of cyanidin-3-(6″malonylglucoside) compared to that of the control was found, with a concentration of 436.6 μg/g DW and preservation of 90%, while cyanidin-3-(3″,6″, dimalonylglucoside) presented 219.8 μg/g DW, which corresponds to 92%. Finally, the bioaccessible fraction obtained after the intestinal phase was 44.4 μg/g DW for Cy-3-Glu, 130.7 μg/g DW for Cy-3-(6″MalGlu), and 57.1 μg/g DW for Cy-3-(3″,6″, diMalGlu) that corresponds to the remaining percentage concerning the control of 17, 27, and 24%, respectively. The three different marker anthocyanin losses accounted for around 80%; despite the degradation of anthocyanins, cyanidin-3-(6″malonylglucoside) remained the most predominant compound among those available for uptake. Important changes in the profile after the intestinal phase were recorded for the marker compounds; in particular, the concentration of each anthocyanin decreased, confirming its high instability during the digestion process.

The results described are in agreement with the data reported by David et al.,^58^ who evaluated the bioaccessibility of Cornelia cherry anthocyanin. Indeed, this work reported that Cornelian cherries’ anthocyanins were stable in the stomach, and the duodenal digestion dramatically decreased the total anthocyanin content and antioxidant capacity levels in the fruit extract, as in our study. Ferron et al.^38^ have shown results that are in line with ours in a new Italian purple maize variety, “Moradyn” flour, finding an increase in the concentration of anthocyanins after the gastric phase and a strong reduction thereafter at the end of the intestinal phase.

The effect of digestion on the content of purple rice anthocyanins was explored by Sun et al.^59^ In contrast with our results, at the end of in vitro gastrointestinal digestion, peonidin-3-glucoside remained the most predominant compound.

As the results and reported data show, anthocyanins are highly unstable and very susceptible to degradation by oxygen, temperature, enzymes, and pH, which are some of the many factors that may affect the chemistry of anthocyanins and, consequently, their stability, color, and molecular structure. As known, anthocyanins are stable in acidic solutions (pH 1–3), and this is important because they are exposed to different pH conditions through the gastrointestinal tract, which affects their bioavailability and hence their bioactivity.^60^ Interestingly, anthocyanins had the highest concentration in the gastric phase of in vitro digestion, which is positive because, in human digestion, anthocyanins could be absorbed in the stomach in their intact form. Moreover, in the intestinal phase, a slight fraction of anthocyanins was found despite their instability at this pH, and this is particularly important because, in the human digestive system, anthocyanins could also be adsorbed intact or could be broken down in the large intestine by the action of the microbiota.^10^

### 3.3 Bioactivity Analysis

#### 3.3.1 Cell Viability Assay

**Figure 4:**
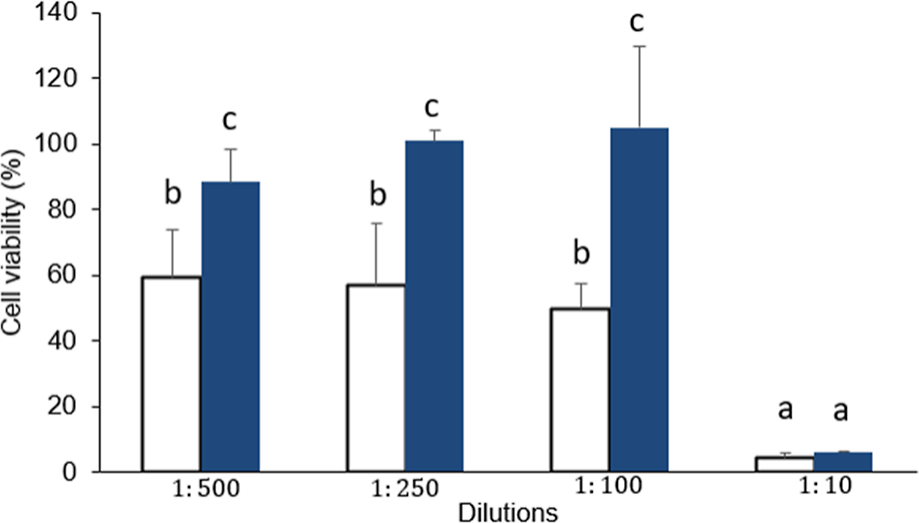
Cell viability assays in Caco-2 of the control (white bars) and intestinal aliquots (blue bars) of Moragro flour. Different letters indicate statistically significant differences in the DGS test (*p* < 0.05).

The viability of Caco-2 cells was evaluated to determine the toxicity of the digested extracts in cell culture and to determine the bioactivity of “Moragro” polyphenols. Figure 4 shows the effect of bioaccessible potential at different dilutions. A significant difference was observed at 1:100 dilution between the intestinal aliquots of “Moragro” flour and the control, with an increase in cell viability of 55.2% in the intestinal aliquot of “Moragro” flour compared to that of the control. At 1:250 and 1:500 dilutions, significant differences were observed with 43.9 and 29.3% of cell viability, respectively. Therefore, the effects found were due to a protective effect provided by the compounds present in the “Moragro” flour since the cytotoxic effect of the control sample remains constant and could be explained due to the reagents involved in the in vitro digestion. Despite the low antioxidant activity at the end of in vitro digestion, the results suggest that the polyphenol compounds remain bioactive and protect the cells.

To our knowledge, there are few studies with the cell model after in vitro digestion of purple maize.^25,61^ Moreover, some of them used an anthocyanin extract, which resulted in much information lost, such as the interaction of polyphenols with matrix compounds and the changes that occur during digestion, and the bioavailability of the compounds has not been well studied. Urias-Lugo et al.^25^ analyzed the antiproliferative activity in different cells of anthocyanins and phenolic acids from blue maize extract. Cell viability of cancer cells (Caco-2) reported viabilities below 30%. Another study was performed on blue maize extracts and evaluated their antiproliferative activity in several cell lines, but in vitro digestion was not performed.^61^

Future perspectives could focus on studying the bioavailability of phenolic compounds and their health effects, considering whole foods.

Overall, “Moragro” maize variety phenols have been characterized in detail. Showing that this purple maize constitutes an excellent source of phenolic compounds, some of them specific for this variety. A total of twenty-six phenols, 15 nonanthocyanins and 11 anthocyanins were found. We showed for the first time the presence of pyrogallol, caffeic acid, kaempferol 3-*O*-glucuronide, kaempferol 3-(6″-feruloylglucoside), citric acid, and gallic acid among nonanthocyanin compounds in purple maize. The most abundant phenol was caffeic acid, representing 22.1% of the total phenols identified. The percentage of anthocyanin compounds was 24.9%, and cyanidin-3-(6″malonylglucoside) was the most abundant anthocyanin. These characteristic compositions of “Moragro” represent a beneficial feature due to the fact that methoxylated derivatives and condensed compounds present higher stability and potential as colorants. In addition, the “Moragro” cultivar showed similarities in proximal composition, anthocyanin profile, and content with other purple maize cultivars, as well as in many nonanthocyanin constituents. This may be due to its direct progenitor varieties as the Argentinean purple maize “Moragro” is derived from varieties descended from Mexico, Peru, Bolivia, and northern Argentina. This is supported by the presence of condensed forms of anthocyanin-flavanol that have not been detected in European varieties to date. “Moragro” flour proved to be an important source of anthocyanins.

The static in vitro digestion method used in our study enabled the quantification of bioaccessible polyphenolic compounds and their antioxidant activity. Despite a relatively high content of bioaccessible polyphenols, low antioxidant activity and only trace amounts of anthocyanins were found at the end of the in vitro digestion. We detected the presence of three major anthocyanins, cyanidin-3-glucoside, cyanidin-3-(6′malonylglucoside), and cyanidin-3-(3″,6″, dimalonylglucoside) at the end of in vitro digestion. Cyanidin-3-(6′malonylglucoside) was the most abundant. This was a positive result since anthocyanins could be absorbed in the gastrointestinal system in their intact form or could be further digested in the gut, serving as a substrate for the gut microbiota. “Moragro” polyphenols showed a protective effect against Caco-2 cytotoxicity. The results suggest that “Moragro” flour is an excellent source of bioaccessible and bioactive compounds that make it a good option as a functional ingredient for the preparation of gluten-free foods with additional healthy properties.

## Data Availability

The data presented in this study are available in the article.
